# The role of FGF21 and its analogs on liver associated diseases

**DOI:** 10.3389/fmed.2022.967375

**Published:** 2022-11-15

**Authors:** Kimia Falamarzi, Mahdi Malekpour, Mobin Fallah Tafti, Negar Azarpira, Mehrdad Behboodi, Mohammad Zarei

**Affiliations:** ^1^Student Research Committee, Shiraz University of Medical Sciences, Shiraz, Iran; ^2^Transplant Research Center, Shiraz University of Medical Sciences, Shiraz, Iran; ^3^Renal Division, Brigham and Women's Hospital, Harvard Medical School, Boston, MA, United States; ^4^John B. Little Center for Radiation Sciences, Harvard T. H. Chan School of Public Health, Boston, MA, United States

**Keywords:** fibroblast growth factor 21 (FGF21), NASH, NAFLD, HCC, MAFLDs, FGF21 polymorphism, FGF21 analogs

## Abstract

Fibroblast growth factor 21 (FGF21), a member of fibroblast growth factor family, is a hormone-like growth factor that is synthesized mainly in the liver and adipose tissue. FGF21 regulates lipid and glucose metabolism and has substantial roles in decreasing lipogenesis and increasing hepatic insulin sensitivity which causing lipid profile improvement. FGF21 genetic variations also affect nutritional and addictive behaviors such as smoking and alcohol consumption and eating sweets. The role of FGF21 in metabolic associated diseases like diabetes mellitus had been confirmed previously. Recently, several studies have demonstrated a correlation between FGF21 and liver diseases. Non-alcoholic fatty liver disease (NAFLD) is the most prevalent type of chronic liver disease worldwide. NAFLD has a wide range from simple steatosis to steatohepatitis with or without fibrosis and cirrhosis. Elevated serum levels of FGF21 associated with NAFLD and its pathogenesis. Alcoholic fatty liver disease (AFLD), another condition that cause liver injury, significantly increased FGF21 levels as a protective factor; FGF21 can reverse the progression of AFLD and can be a potential therapeutic agent for it. Also, NAFLD and AFLD are the most important risk factors for hepatocellular carcinoma (HCC) which is the fourth deadliest cancer in the world. Several studies showed that lack of FGF21 induced oncogenic condition and worsened HCC. In this review article, we intend to discuss different aspects of FGF21 in NAFLD, AFLD and HCC; including the role of FGF21 in pathophysiology of these conditions, the effects of FGF21 mutations, the possible use of the FGF21 as a biomarker in different stages of these diseases, as well as the usage of FGF21 and its analog molecules in the treatment of these diseases.

## Introduction

Fibroblast growth factor 21 (FGF21) is an endocrine regulating factor that is produced mainly in the liver and adipose tissues (1, 2). FGF21 can improve many critical liver-associated diseases by contributing to metabolic pathways. Reducing lipogenesis, inducing fatty acid β-oxidation, increasing hepatic insulin sensitivity, decreasing very-low-density lipoprotein (VLDL) transmission to the liver and subsiding the hepatic endoplasmic reticulum (ER) stress are the major mechanisms of FGF21 to improve fatty liver diseases (3–5). It is also reported that FGF21 could be a protective factor against lipotoxicity (1). Furthermore, FGF21 can induce insulin sensitization and increase the glucose uptake in white adipose tissues (1, 3, 4, 6). In addition, FGF21 may reduce the risk of atherosclerosis due to lowering inflammation, regulating of lipid metabolism and its effect on adiponectin expression (7).

The liver is one of the most important organs in the body because of its crucial role in several processes including detoxification, anabolism and catabolism, immune factors production and lipids metabolism regulation. The liver's functions can be affected by several diseases. Non-alcoholic fatty liver disease (NAFLD) is the most common chronic liver disease worldwide. Insulin resistance, lipid metabolism dysfunctions and inflammation are the major causes of NAFLD (8). Decreased mitochondrial fatty acid oxidation, increased hepatic lipogenesis and decreased lipid export from hepatocytes are the mechanisms that may lead to hepatic steatosis (9). NAFLD is defined by fatty infiltration of the liver in more than 5% of hepatocytes and in 20% of patients progressed from Nonalcoholic Steatohepatitis (NASH) to liver fibrosis and eventually cirrhosis (10, 11). Patients with NAFLD and NASH have a high risk for developing cardiovascular diseases, cirrhosis and hepatocellular carcinoma (HCC) (12, 13). Some studies found an association between FGF21 levels in serum and the amount of fatty contents in the liver (14). Therefore, FGF21 can be used as a diagnostic biomarker for NAFLD (8, 14). FGF21 can modulate oxidative and ER stress, decrease fat synthesis and the levels of inflammatory cytokines (15–18) and enhance the expenditure and catabolism of stored lipids (19).

NAFLD is the leading risk factor for HCC (20). HCC is the most common primary liver cancer which is fatal due to its late diagnosis (21, 22). The 5-year average survival rate of HCC is <10% (22). Because FGF21 rise at an early stage of HCC, it can be used as a diagnostic factor for HCC (23–25). However, the FGF21 levels were decreased when HCC is well-developed (26, 27). Lack of FGF21 can accelerate the progression of NAFLD to HCC *via* induction of inflammation and accumulation of lipids in the liver (28, 29). Overexpression of FGF21 likely delays the development of adenomas at an early stage of carcinogenesis (30).

FGF21 protects the liver not only from NAFLD and NASH but also from alcoholic fatty liver disease (AFLD). About 50% of cirrhosis-related death is attributed to alcohol consumption (31). Chronic alcohol consumption may lead to the accumulation of lipids in hepatocytes and liver injury (32). Previous studies show that alcohol usage can increase FGF21 serum levels (33). FGF21 may ameliorate AFLD by improving hepatomegaly, reducing lipid synthesis, enhancing mitochondrial oxidative function and decreasing the production of reactive oxygen species (32–35).

In this review article, we attempt to concisely explain the role of FGF21 and its mutations and analogs on liver disorders. Further studies will be required to determine the effectiveness and accuracy of FGF21 and its analogs in targeted therapy to cure and diagnose hepatic disorders.

## FGF21

### FGF21 mechanism of action

FGF21 is a hormone-like growth factor composed of 209 amino acids (1). In humans, FGF21 (gene ID: 26291) is on chromosome 19 (19q13.33) and contains 3 exons that encode this protein (36). FGF21 physiology is somewhat complex mainly because it is secreted from different organs and affects various organs (36). FGF21 is secreted predominantly from the liver and adipose tissues (1, 2) even though there are many other sites in which FGF21 is synthesized such as the pancreas, skeletal muscles and cardiac endothelium (3, 37). FGF21 can be released from the site where synthesized into the bloodstream to act as an endocrine hormone (4). FGF21 is also detectable in human cerebrospinal fluid (2, 5). FGF21 binds to fibroblast growth factor receptors (FGFRs) with extremely low affinity and since it lacks a heparin-binding domain, the presence of a co-receptor called β-klotho is required for improving the affinity of FGF21 binding (1, 3, 36). β-klotho is a transmembrane protein that is necessary for FGF21 signaling and its activities on target tissues (1, 36). β-klotho is expressed in specific metabolic tissues such as the liver, pancreas and adipose tissues which determines the FGF21 target organs while FGFRs are expressed in various tissues and cells such as kidney, liver, adipose tissues, skeletal muscle and etc but mainly in liver and adipose tissue (1, 3, 13). In addition, hepatic FGF21 expression is strongly controlled by peroxisome proliferator-activated receptor α (PPARα), a transcription factor activated by the non-esterified fatty acids released from adipocytes which decreases the lipogenesis and increases fatty acid β-oxidation (38).

Several studies confirmed that FGF21 has an important role in lipid and carbohydrate metabolism as well as energy and nutrient homeostasis (39). Hence, FGF21 could be considered a potential diagnostic biomarker and therapeutic agent for metabolic diseases such as obesity, type 2 diabetes mellitus (T2DM), and fatty liver (4).

FGF21 increases hepatic insulin sensitivity, decreases lipogenesis, triggers fatty acid β-oxidation, reduces hepatic ER stress, and diminishes VLDL delivery to the liver (through down-regulation of VLDL receptor expression in hepatocytes) (3–5). FGF21 could also decrease postprandial triglycerides (TGs) and facilitate fatty acid storage in adipose tissue (40). These actions eventually result in lipid profile improvement, weight loss, decreased hepatic triglyceride content, and ameliorate fatty liver, NASH, and metabolic-related diseases (3, 39).

FGF21 levels are positively correlated with obesity, body mass index (BMI) and hepatic fat accumulation (1, 36). It is demonstrated that increased FGF21 levels could be an adaptive mechanism to protect the body against lipotoxicity (1). Several studies argued that FGF21 administration increased the browning of white adipose tissue and activation of brown adipose tissue; therefore resulting in increased energy expenditure, maintained body temperature during cold exposure and ultimately weight loss (4, 41).

FGF21 level increased in T2DM and is positively correlated with hyperglycemia, insulin resistance and inflammatory processes (1). FGF21 increased glucose uptake in white adipose tissue through induction of glucose transporter1 (GLUT1) expression which is and independent process from insulin (3, 4, 6). Previous studies demonstrated that FGF21 could lead to a rapid insulin sensitization within 1 hour (1, 3). Administration of FGF21 or its analogs significantly increased plasma adiponectin levels (1, 3, 4). Adiponectin is an insulin-sensitizing factor which mainly secreted from adipocytes and has anti-inflammatory and anti-sclerotic effects and it mediates FGF21 impacts on energy metabolism and insulin sensitivity (4, 38). FGF21 increased adiponectin gene expression. The secretion of adiponectin is from adipocytes through a Peroxisome proliferator-activated receptor γ (PPARγ)-dependent mechanism (4). PPARγ is a transcription factor that its activation increases FGF21 effects such as reduction of fat and lipidemia, improvement of tissue insulin sensitivity and increase of lipogenesis in white adipose tissue (1, 5).

In contrast to mouse models, short-term fasting or ketogenic diets do not increase FGF21 levels in humans (2). Long-term starvation (about 7–10 days), high sugar intake and protein restriction in diet cause FGF21 elevation in humans (3, 42). FGF21 mutations also are associated with macronutrient preference in humans independently of BMI (2, 6, 43). These mutations are believed to have associations with increased sweet taste preference in humans (3). Further studies showed that, the administration of an FGF21 analog to obese individuals can decrease the preference for sweet-tasting food and carbohydrate intake (3, 44).

FGF21 also is elevated in patients with atherosclerosis (1, 7). Several studies indicated that FGF21 by its anti-oxidative and anti-inflammatory effects and its influences on lipid profile and adiponectin expression could, directly and indirectly, decrease atherosclerosis incidence (7).

**Figure 1:**
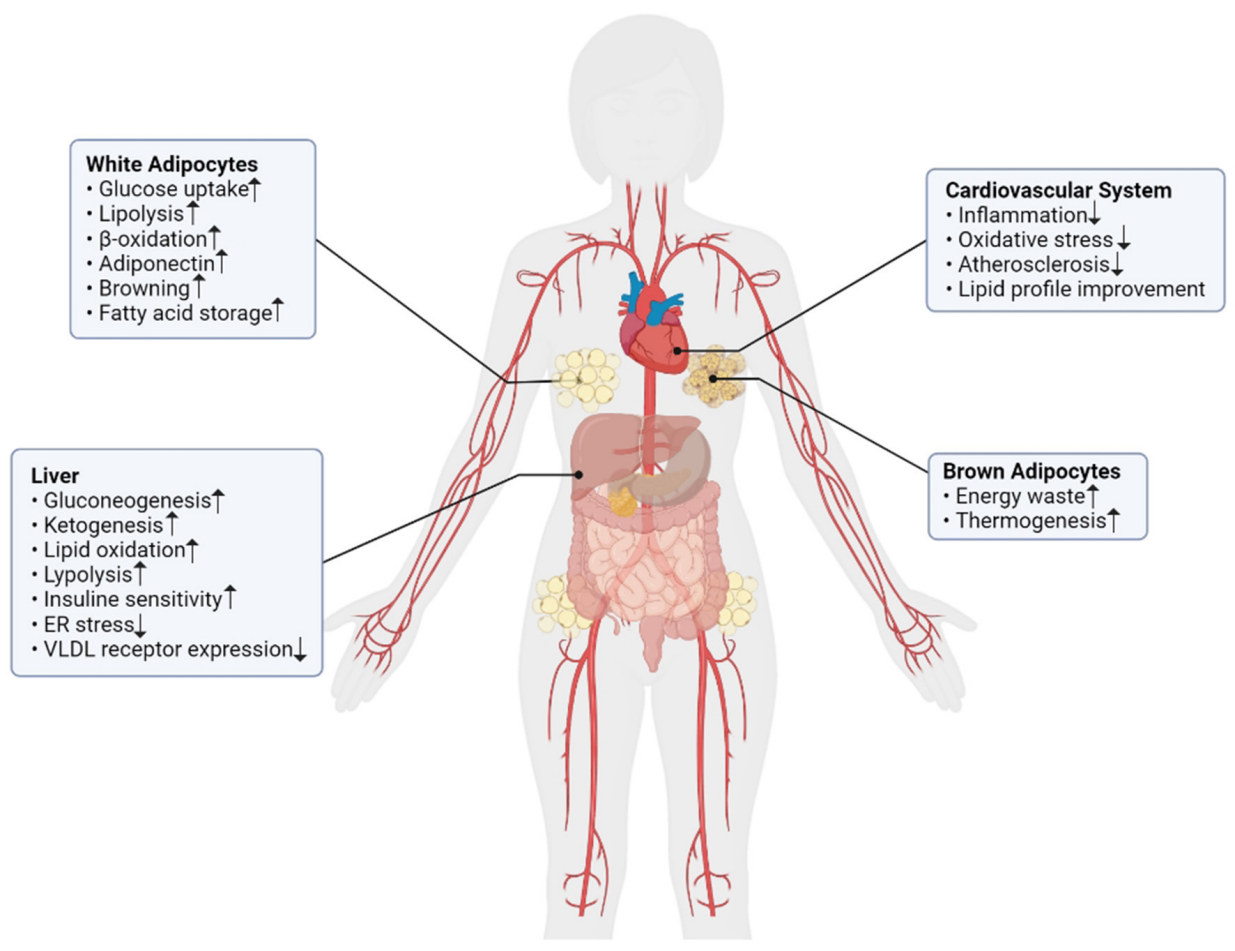
The summarized effects of FGF21 in different metabolic pathways of body.

These findings suggested that FGF21 increased in obesity and other related conditions such as T2DM, metabolic syndrome, fatty liver disease, etc. FGF21 has several important roles in lipid and carbohydrate metabolism and energy homeostasis which it's important roles summarized in Figure 1. Considering these facts, FGF21 could be a potential agent for the diagnosis and treatment of metabolic-related diseases. Further studies will be required to determine its effectiveness and accuracy.

### FGF21 genetic variations

To the best of our knowledge, there are not enough studies about the association of genetic variations of FGF21 with hepatic diseases such as NAFLD, NASH, and HCC. While FGF21 can affect the pathomechanism of these diseases, it can also play an important role as a biomarker for NAFLD and AFLD and show significant results in curing fatty liver and metabolic associated diseases. In a study done on a Han Chinese non-diabetic population the association of four Single Nucleotide Polymorphisms (SNPs) with NAFLD was investigated (45). They found that rs499765 was associated with serum levels of FGF21 and can be a predicting factor for measuring the risk of NAFLD (45). Besides, they found that rs2071699 and rs838136 correlate with aspartate aminotransferase serum concentration and rs838136 is associated with alanine aminotransferase levels (45).

Another study was done in order to discover the association between genetic variations of FGF21 and Metabolic Associated Fatty liver diseases (MAFLDs) suggesting that rs838136 could be a risk factor for MAFLDs *via* changes in folding and stability of FGF21 mRNA (46).

Furthermore, previous research found that rs838133 is correlated with behaviors such as alcohol and candy consumption and also daily smoking. This study implies that the liver can regulate eating and lifestyle habits *via* producing different hormones like FGF21 (47). The other studies confirmed the role of FGF21 SNPs in addictive behaviors like eating habits and the amount of coffee consumption (48–51). Previous studies also showed the importance of FGF21 genetic variations in obesity (rs11665896) (50), fat and macronutrient intake (rs838147) (51), renal function in diabetic patients (rs2071699, rs838136, and rs499765) (52), and alcohol dependence (rs11665896) (53) which indirectly can have effects on steatosis of liver.

Earlier studies also have suggested that FGF21 serum level could be a biomarker for dysregulated metabolic pathways and also the level of fat accumulation in the liver (14, 54). This upregulation of FGF21 could also help to prevent fat deposition in the liver resulting in reduced inflammation and fibrosis of the liver (55). Moreover, the results of a cohort study demonstrated that a higher serum level of FGF21 could be a prognostic factor for HCC (23). Consequently, due to the importance of circulatory FGF21 concentration the SNPs regulating the serum level of FGF21 are considerably crucial in liver diseases. Previously a Genome-Wide Association Study (GWAS) reported the most important SNPs which regulate FGF21 serum levels. These SNPs were rs12565114, rs9520257 and rs67327215 (56). Investigations on the role of these SNPs and the susceptibility of people to having liver diseases may help to personalized the cure of these diseases.

At last, according to the significant role of the FGF21 gene in metabolic associated diseases and behavioral habits related to these diseases and additionally the direct influence of FGF21 variations on MAFLDs as well as the heritability pattern of fatty liver diseases (57, 58), there should be further studies for investigating the association of FGF21 genetic variations and MAFLDs.

## The relation between FGF21 and liver associated diseases

### FGF21 in alcoholic fatty liver disease

Alcohol-related deaths have the third rank among the most common preventable causes of death after smoking and hypertension (59). More than 200 diseases and a range of injuries have a link to the consumption of alcohol, such as cardiovascular diseases, cirrhosis, and several cancers (60). Liver diseases particularly cirrhosis has the largest alcohol-attributable fraction; Almost 50% of cirrhosis-related mortality is caused directly or indirectly by alcohol (31). In 2010, almost half a million deaths occurred due to alcohol-related cirrhosis (61).

Alcoholic liver diseases consist of a spectrum of pathologies ranging from alcoholic hepatitis to cirrhosis, and cirrhosis complications (2). AFLD is one of the major causes of mortality in the United States, with nearly 250,000 deaths due to AFLD in 2010 (62).

Among the risk factors of AFLD, the amount and duration of an individual's alcohol consumption are the most important factors (60). Also, it has been demonstrated that gender is another risk factor for AFLD because the relative risk of AFLD is higher in women than men (63). Also, chronic viral hepatitis such as hepatitis C and genetic and epigenetic factors have been suggested as the risk factors for AFLD (60, 64).

Accumulation of alcohol in hepatocytes as a result of chronic alcohol consumption can induce liver injury (32). Alcohol-induced fatty liver injury is reversible at the initial stages but AFLD can develop more severe forms of liver injury such as alcoholic hepatitis, cirrhosis, and hepatocellular carcinoma as long as the individual continues alcohol consumption (32). Alcohol also affects the liver through nutritional disturbance as a result of its metabolism process in the liver (65).

Acute alcohol consumption increases FGF21 levels in both humans and mice (66). Previous studies demonstrated that FGF21 levels significantly increased more than 3 fold by acute ethanol intake (67) and alcohol exposure lead to increased hepatic FGF21 expression and its circulating level (68). Additionally, patients with alcoholic steatohepatitis had FGF21 levels 6 times greater than non-drinking healthy subjects without any liver diseases (68). Chronic alcohol exposure results in FGF21 up-regulation in mice and the absence of FGF21 causes substantial liver pathologies (69). Chronic alcohol consumption could cause hepatic lipogenesis and impaired fatty acid β-oxidation by hepatic factors dysregulation such as PPARα, Sirtuin 1, and Adenosine monophosphate-activated protein kinase (AMPK) (68).

Adenosine monophosphate-activated protein kinase (AMPK) is a metabolic regulator which senses the oxidative stress and reduced energy charge of body. Several energy-generating pathways such as glycolysis and fatty acid oxidation are up-regulated by AMPK (32). AMPK also inhibits the activity of several energy-demanding processes, including fatty acid, cholesterol, and protein synthesis (32). Several studies have demonstrated that the activity of AMPK is decreased in ethanol-fed rodents (70, 71), as a result, fatty acid synthesis is promoted in these rodents, whereas the fatty acid oxidation pathway is blocked (71). In conclusion, the pathogenesis of AFLD is associated with AMPK inactivation (32). FGF21 also regulates energy homeostasis in adipocytes by activating AMPK and Sirtuin 1, which results in enhanced mitochondrial oxidative function (35). Intracellular reactive oxygen species production induced by alcohol in hepatic cells can remarkably decrease by FGF21 (32).

Besides, alcohol exposure may lead to hepatic fat accumulation, hepatic ER stress and inflammation which results in FGF21 production (68). Several studies demonstrated that FGF21 expression induced by alcohol is a hepatic adaptive response due to lipid dysregulation (68). Lipid synthesis can be inhibited due to an increase in FGF21 serum levels (72). The mRNA expression of lipogenic genes, such as fatty acid synthase (FAS) and acetyl-CoA carboxylase 1 and 2 (ACC1 and ACC2) are significantly suppressed by FGF21 (73). The role of FGF21 in ameliorating lipid metabolism has also been demonstrated (73). The rise in FGF21 expression leads to increased fatty acid oxidation and limited lipid accumulation (69). Loss of FGF21 in mice leads to worsening of alcohol-induced steatohepatitis and liver injury which is due to increased activation of genes involved in lipogenesis and decreased expression of genes involved in fatty acid oxidation (68). Also, FGF21 Knocked Out mice showed an enhanced hepatic inflammation due to alcohol exposure (68, 74). These results suggested that, the protective effects of FGF21 in alcoholic liver disease might be associated with de novo lipogenesis and fatty acid catabolism and also its role as an anti-inflammatory factor (2, 68). Some studies showed that FGF21 administration could ameliorate alcoholic liver disease in mice (32, 68). These studies suggested that FGF21 had positive effects on serum lipid profile, decreased hepatocytes lipid accumulation and reduced oxidative stress in mice with alcoholic fatty liver disease (32). Additionally, FGF21 related treatments could prevent fatty liver progression and reverse the development of AFLD in mice (68). The results of the experiments on the plasma of ethanol-fed rodents have demonstrated a significant increase in FGF21 serum level, therefore alcohol consumption can increase FGF21 gene expression (72). FGF21 also plays a preventable role against hepatomegaly and can reduce the swelling of the liver, which can improve AFLD (32).

According to these findings, FGF21 might be a potential target for AFLD treatment and further trials are required to investigate its effects as a therapeutic agent in humans.

### FGF21 in NAFLD and NASH

Non-alcoholic fatty liver disease is nowadays the most common chronic liver disease in the world. It affects 1.8 billion people worldwide with a prevalence of about 30% of the adult population (75, 76). NAFLD is the main risk factor for the development of hepatocellular carcinoma. NAFLD is also associated with obesity, diabetes mellitus and metabolic syndrome and it can be a hepatic manifestation of metabolic syndrome (18). NAFLD is defined as the accumulation of lipid in more than 5% of total liver weight when there are no secondary causes such as excessive alcohol intake, infections, autoimmune diseases, or any other etiologies of liver diseases (8). The comparison table between different aspects of AFLD and NAFLD can be seen in Table 1.

**Table 1:** The comparison table between NAFLD and AFLD.

	**NAFLD (77)**	**AFLD (78, 79)**
Prevalence	30% (About 1 billion people worldwide)	4%
Risk factors	Diabetes, obesity, metabolic syndrome, age, male gender	Amount and duration of alcohol consumption, genetic factors, female gender
Pathophysiology	Insulin resistance, hepatic fat accumulation, inflammation	Hepatic fat accumulation, hepatic ER stress, inflammation
Complications	NASH, cirrhosis, HCC	Chronic fibrosis, cirrhosis, HCC
Treatment	No specific pharmaceuticals are currently FDA approved	Cessation of alcohol consumption, no specific medical treatment approved
FGF21	Elevated expression and serum FGF21	Elevated expression and serum FGF21

The pathophysiology of NAFLD is still unknown; however, insulin resistance, lipid metabolism dysfunctions and inflammation are established as the main pathogenic pathways for developing NAFLD (8). Steatosis occurs when there is an imbalance between the input and export of hepatocellular fat. The sources of hepatic fat can come from dietary intake, fatty acid flow to the liver from adipose tissue and hepatic *de-novo* lipogenesis (9). Insulin resistance is another important factor for lipogenesis in NAFLD. Other mechanisms that cause hepatic steatosis are decreased mitochondrial fatty acid oxidation, increased hepatic lipogenesis and decreased lipid export from hepatocytes (9). Fat accumulation can lead to lipotoxicity, oxidative stress, immune cell, and satellite cell activation which result in hepatic inflammation and fibrosis (9).

The pathological spectrum of NAFLD ranges from simple hepatic steatosis to non-alcoholic steatohepatitis and hepatic fibrosis and cirrhosis (15, 76). Even simple steatosis can put individuals at risk for developing NASH (8). NASH has a global prevalence of 1.5–6.5% in adults and patients with NASH have an increased mortality rate compared to the general population due to an increased rate of cardiovascular diseases, cirrhosis, and hepatocellular carcinoma (12). NASH is currently the second leading cause of cirrhosis among adults who are waiting for liver transplantation. About 20% of NASH patients develop liver cirrhosis (11). The inability of hepatocytes to regulate fatty acids overload may lead to NASH development. Fatty acid excess can cause lipotoxicity, mitochondrial dysfunction, ER stress, activation of inflammatory pathways, cell injury and cell death. Those changes will eventually induce fibrosis, cirrhosis and HCC (80). Inflammation and cell injury are important factors that define NASH and when these mechanisms took place, simple steatosis turns into NASH (17).

FGF21 has significant roles in lipid and glucose metabolism and energy homeostasis. Single nucleotide polymorphisms of FGF21 were associated with the pathogenesis of NAFLD (15). Previous studies found that serum FGF21 was elevated in NAFLD and significantly correlated with hepatic fat content (or intrahepatic TG content) (14). Consequently, FGF21 could be a potential diagnostic marker for NAFLD (8, 14). A meta-analysis published in 2017 suggested that FGF21 showed excellent performance to distinguishing NASH from hepatic steatosis and it performed well in identifying NASH; however, its ability to confirm the diagnosis was inadequate due to the fact that the number of studies included was very few (this biomarker was modestly sensitive and specific, with pooled values of 62 and 78%, respectively) (81, 82). Another study suggested that FGF21 had sensitivity and specificity of 72.6 and 85.1% for diagnosis of NAFLD as well as sensitivity and specificity of 53.7 and 71.9% for diagnosis of NASH (83).

Free fatty acids stimulated FGF21 expression *via* PPARα activation (19, 76, 84). In previous human studies a significant association of both serum concentration and liver mRNA expression of FGF21 with hepatic fat and TG content was found (76). Previous investigations demonstrated that FGF21 levels increased in mild and moderate NAFLD patients but as hepatic fat content increased and severe NAFLD occurred FGF21 concentration decreased. This happened due to hepatic cell injury or death caused by lipotoxicity and hepatic inflammation in severe NAFLD or NASH patients so that the remaining hepatic cells were unable to produce as much FGF21 as needed (54, 76, 85). Accordingly, FGF21 might be sensitive in diagnosing mild or moderate steatosis and predicting the onset of simple steatosis (76). While for severe NAFLD and NASH diagnosis combining FGF21 with other circulating markers like cytokine 18 (CK-18) seems more preferable with an overall specificity of 95% and a positive predictive value of 90% (2). Hence, FGF21 is better for predicting the onset of simple steatosis, while other markers (such as CK-18) are better for predicting the prognosis of NAFLD (2).

FGF21 is one of the most potent insulin sensitizers (3) and since insulin resistance is one of the most important factors in NAFLD development, FGF21 with this mechanism can ameliorate NAFLD (18). Besides, intact insulin signaling is necessary for most of the FGF21 effects on lipid metabolism (16).

FGF21 also modulates the process of oxidation stress, ER stress, mitochondrial dysfunction and inflammation to slow the progression of NAFLD (15). FGF21 level increase in NAFLD, in order to sustain homeostasis against lipotoxicity, oxidative stress, and ER stress (14). FGF21 decreased gene expression related to fat synthesis such as FAS, acetyl ACC1 and ACC2, and significantly increased gene expressions related to energy expenditure (18). *In vitro* experiences also revealed a crucial role of FGF21 in fat metabolism and hepatic lipid regulation (10). These functions resulted in decreasing lipogenesis, increasing lipolysis of lipid droplets, the clearance of fatty acids, and the enhanced expenditure of the stored lipid energy by enhanced mitochondrial substrate oxidation, catabolism and uncoupling (19). Previous studies found that FGF21 analogs can also improve mitochondrial functions in a way that mitochondria could manage excessive fatty acids without producing reactive oxygen species (17). Preclinical studies have demonstrated that FGF21 has anti-inflammatory, anti-diabetic and hypolipidemic roles. Therefore, the administration of FGF21 analogs has been shown to reverse hepatic steatosis in both mice and humans (8). In NASH mouse models using leptin-deficient mice and methionine and choline-deficient diet, FGF21 analogs reversed hepatic inflammation and fibrosis (15–17). It can also reduce hepatic inflammation and immune cell infiltration in mice (8). Inflammation can suppress β-klotho expression and impair FGF21 signaling leading to FGF21 resistance (8). The elevation of FGF21 levels observed in individuals with NAFLD is likely due to a compensatory response to FGF21 resistance. FGF21 also reduced the level of the inflammatory cytokines such as Interleukin-18 (IL-18) and Tumor necrosis α (TNF-α) (16, 17). Autophagy is an important mechanism in recycling cytoplasmic components and damaged organelles (86). Defective hepatic autophagy results in abnormal accumulation of hepatic TG, insulin resistance, fatty liver and ultimately more serious hepatic conditions like HCC (18). Hepatic expression of autophagy components and autophagy gene activators are decreased in NAFLD patients (86). FGF21 significantly increases the expression level of genes related to autophagy (18, 86).

In the end, considering FGF21 roles in glucose and lipid metabolism and also in energy balance and according to its effects on NAFLD and NASH, FGF21 could be a potential biomarker for diagnosis of NAFLD and NASH and it might also be a target for the treatment of these conditions. Hence, further studies and trials are needed to identify FGF21 and its mutations' roles in NAFLD and NASH development and also FGF21 analogs' effectiveness in NAFLD and NASH treatment.

### FGF21 in HCC

HCC is the most common primary liver cancer which is the fifth most common cancer in men and the seventh in women (21, 87). HCC is the 4th deadliest cancer in the world and its mortality and morbidity rates have been increasing over the past decades (21, 88, 89). Also, HCC has a very poor prognosis because of the late diagnosis of the disease. The 5-year average survival rate of HCC is <10% (22). Therefore, studying the risk factors and molecular mechanisms of HCC can continue the progression of understanding the disease.

HCC is a multi-stage cancer that is induced by many factors. Viruses, aflatoxin, alcohol usage, lack or mutations in some regulatory genes such as FGF21 and many other factors that cause hepatic injury, can stimulate a preceding process of HCC (90). Hepatic injury may trigger the proliferation and regeneration of hepatocytes (90). The following hepatic inflammation and the presence of several cytokines, growth factors, chemokines and oxidative stress components play a major role in making an environment wherein hepatocytes can alter phenotypically (90). Also, NAFLD and NASH are the leading causes of HCC (20). Fatty acids concentrating in the liver can induce steatosis and inflammation. Inflammatory cytokines and infiltrating macrophages may cause chronic inflammation and liver cells death subsequently. These reactions along with some factors such as transforming growth factor β (TGF-β) and IL-18 extremely increase the risk of liver cancer (20). In addition, the nodules that appeared in cirrhosis also can provide a condition that transforms the normal hepatocytes into dysplastic hepatocytes (90). Hepatocyte injury, inflammation and proliferation of liver cells and subsequent fibrosis and cirrhosis predispose the liver to cancer.

FGF21 is a liver-derived factor that regulates lipid and glucose metabolism (38, 91). Lack of FGF21 could induce inflammation and lipid accumulation in the liver (29). In the absence of FGF21 the production of Interleukin-17A (IL-17A), a critical factor for NASH development, is highly increased (29). IL17-A recruits macrophages and neutrophils into the inflammation area (29). In addition, FGF21 reduces lipid concentration in the liver by activating of sirtuin 1 pathway and preventing lipolysis (28, 29). FGF21 also regulates the inflammatory cytokines due to its negative impact on the NF-κB (nuclear kappa light chain enhancer of activated B cells) mediated TGF-β signaling pathway (92).

Some viruses such as Hepatitis C Virus (HCV) and Hepatitis B Virus (HBV) can be major risk factors for HCC (93). FGF21 is a novel diagnostic biomarker to monitor the progression of chronic hepatitis B (CHB) (24) and chronic hepatitis C (CHC) (94, 95). Also, Obesity and diabetes mellitus have a crucial role in developing HCC (21). A study reveals that exogenous FGF21 can decrease blood glucose and serum triglycerides to the normal amount in obese or diabetic mice (96). Alcohol intake also can be a contributing factor to liver cancer (21, 93). Alcohol consumption can increase FGF21 levels (66, 68). The role of diet in HCC is still controversial. Some studies reported that foods containing milk, wheat, vegetable, fish and fruit have reduced the risk of HCC but other studies disclosed no association (21). However previous studies report the role of FGF21 in human diet preferences (48–51).

To the best of our knowledge, few studies reported an association between HCC and the expression of FGF21. FGF21 is induced by liver injury and stress and can be a prognostic biomarker to monitor the carcinogenesis of the liver and established as an early diagnostic biomarker for HCC (23–25). A study reported that higher levels of FGF21 related to worse survival in HCC patients (23). Finn et al. discovered that higher levels of FGF21 associated with shorter overall survival in HCC patients regardless of treatment (97) and they suggested that FGF21 might be an independent prognostic factor for overall survival in HCC (97). P53 is a transcription factor that controls FGF21 expression in some abnormal hepatic functions. P53 is a stress regulator that decreases FGF21 expression in hepatic cells. Also, a study reported that the haploinsufficiency of p53 can progress to carcinogenesis and has a significant effect on increasing FGF21 expression (25). This study showed that the FGF21 levels are significantly increased before the HCC becomes clinically obvious (25). Besides, Liang et al. demonstrated that CHB patients who developed HCC experienced elevated levels of FGF21 (24). Also, another study mentioned that FGF21 was increased in liver cancer and regeneration after partial hepatectomy in a genetic model mouse (30). Although overexpression of FGF21 delays the emergence of adenomas at early stages *via* activating of hepatocyte FGFR4, it accelerates the progression of tumors to HCC by interacting with FGFR1 (30).

The deficiency of FGF21 appears to have a role in the progression of NAFLD to HCC (29, 98). Zhang et al. first demonstrated that diminished FGF21 levels were associated with cancerous hyper-proliferation and atypical oncogening signaling in the liver (27). It appears that the level of FGF21 indicates the amount of triglyceride accumulation in the liver (26, 98). In a study by Garima et al. FGF21 deficient mice were found to have significantly more accumulation of hepatic lipids in comparison with the wild type (WT) mice with the same high fat, high sucrose (HFHS) diet (98). The sinusoidal fibrosis which can develop to HCC was significantly higher in FGF21 KO mice than WT mice with the same diet (98). 78% of FGF21 KO mice on HFHS diet in comparison to 6% of WT mice represent 1–3 large liver nodules which can lead to HCC histologically. Remarkably, HCC was developed without cirrhosis in their study (98). Another study exhibited that lack of FGF21 could worsen the metabolic disorders in NASH and provide the microenvironment, wherein inflammation, regenerating proliferation of hepatocytes and fibrosis may happen (26). This condition which contains many inflammatory and mitotic factors has a high risk to progress to HCC in diabetes mice (26).

A study identified that FGF21 levels were increased at the early stage of hepatic stress in a genetic model mouse presenting diabetes-steatohepatitis (27); however, the reduction in FGF21 levels was reported when HCC was well-developed. This may show that the early rise in FGF21 expression can indicate its protective role of it and the late decrease in expression of FGF21 may refer to chronic hepatic disorders comprising liver cancer (26, 27). Three reasons for the downregulation of FGF21 have been suggested before. First, the expression of the FGF21 gene has a negative association with the concentration of liver triglyceride. Because of the major role of high hepatic lipid concentration in HCC development, FGF21 levels were reduced in carcinogenesis. Second, G9a, a factor that suppresses FGF21 expression epigenetically, modifies the process of HCC. Lastly, hypoxia can reduce the FGF21 mRNA level (99); because of the hypoxic condition of the most solid tumors, the FGF21 levels can be decreased (27). Unlike the early rise of FGF21 in a pre-cancerous liver, due to the lipid accumulation in the liver, the G9a factor and the hypoxic condition of the liver, the level of FGF21 is decreased in well-developed HCC.

In conclusion, FGF21 has a protective and diagnostic role at the early stage of HCC. FGF21 can delay the conversion of adenomas to malignant tumors by regulating inflammation and lipid concentration in the liver. Note that the level of FGF21 seems to be decreased at the late stage of carcinogenesis. This reduction may probably relate to the deteriorating effect of FGF21 on the progression of HCC at an advanced stage of tumorigenesis. Further studies will be needed to find the exact role of the FGF21 in liver cancers.

## FGF21 as a drug

According to the important effects of FGF21 on metabolic associated diseases some clinical trials have been established in order to assess the safety and therapeutic efficacy of human FGF21 analogs and FGF21 receptor agonists (4). Nevertheless, there were some challenges in the use of FGF21 as a drug such as its poor pharmacokinetic characteristics including short half-life (0.5–2 h), poor instability and bioavailability (80, 100). This resulted in the development of FGF21 analogs applying polyethylene glycosylation (PEGylation) or fusion to antibody fragments (80). The summarized table of the FGF21 analogs and clinical trials can be seen in Table 2. In these studies different effects of FGF21 analogs were discussed such as glycemic and lipid profile of blood, fibrosis reduction of livers (*via* Pro-C3 biomarker reduction measurement) and bodyweight.

**Table 2:** The summarized table of the FGF21 analogs and the effect of the analogs on the body.

	**Study groups**	**Hepatic fat content**	**Hepatic inflammation/ fibrosis**	**Blood tests**	**Body weight**	**Reference**	**FGF21 analog**
1	AFLD mice	Suppress hepatocyte lipid droplet accumulation	Decrease oxidative stress	Decreased TG, TChol, LDL	–	Zhu et al. (32)	Recombinant FGF21
2	Alcohol-treated HepG2 cells	Reduced fat accumulation	Suppressed intracellular ROS products	–	–	Zhu et al. (32)	Recombinant FGF21
3	Alcohol-fed Wild type mice	Decrease hepatic fat accumulation	Decrease inflammation	Reduced ALT, AST and plasma TG	–	Liu et al. (68)	Recombinant FGF21 (rhFGF21)
4	Obese cynomolgus monkeys	–	–	Decrease TG- increase adiponectin	Decreases Body Weight and Food Intake	Saswata Talukdar (34)	PF-05231023
5	Obese/overweight humans withT2DM	–	–	Decrease TG and LDL- increase HDL and adiponectin	Decrease body weight	Saswata Talukdar (34)	PF-05231023
6	Obese and T2DMpatients	–	–	Decrease in LDL, TG and increase in HDL and adiponectin	Decrease body weight	Gaich G,2013 (101)	LY2405319
7	Diet-induced obese (DIO) mice	–	–	Decrease plasma glucose	Decrease body weight	Kharitonenkov et al. (102)	LY2405319
8	Patients with biopsy-confirmed NASH	Significant decrease in absolute hepatic fat fraction	Decrease Pro-C3	Decrease ALT, AST, TG and LDL- increase HDL and adiponectin	No substantial changes in bodyweight	Sanyal et al. (12)	Pegbelfermin
9	Patients with obesity and T2DM	–	Decrease Pro-C3	Decrease TG, increase in HDL and adiponectin	No statistically significant decrease in weight	Charles et al. (103)	Pegbelfermin
10	Patients with NASH	Significant reduction in hepatic fat fraction	Decrease Pro-C3	Decrease in AST, ALT, ALP, HbA1C, fasting glucose, cholesterol and LDL- increase HDL and adiponectin	Decrease body weight	Harrison et al. (104)	Efruxifermin
11	Patients with obesity and NAFLD	Decrease hepatic fat content	Decrease Pro-c3	Decrease TG, LDL- increase HDL and adiponectin	Transient body weight reduction	Negi et al. (105)	BFKB8488A
12	Obese cynomolgus monkey	–	–	Increase adiponetin	Decrease weight and food intake	Baruch et al. (44)	BFKB8488A
13	Insulin resistant, obese subjects with increased liver fat	Decrease hepatic fat content	–	Decrease HbA1C, TG, LDL- increase HDL	–	Depaoli et al. (106)	MK-3655,
14	Obese and mildly hypertriglyceridemic adults	Decrease hepatic fat content	Decrease Pro-C3 and improved liver fibrosis	Decrease in AST, ALT, ALP, cholesterol and LDL-increase HDL	–	Rader et al. (107)	LLF580
15	Mice on methionine and choline-deficient diets	Decrease hepatic TG	Decrease inflammation and fibrosis	Decrease in ALT and free fatty acid	No change in body weight	Fisher et al. (108)	Recombinant FGF21

LY2405319, a glycosylated FGF21 variant, in a randomized, double-blind, placebo-controlled study demonstrated significant improvements in lipid profile, body weight and adiponectin level in obese and T2DM patients (101). The main effects of LY2405319 treatment were reduction in low-density lipoprotein (LDL) cholesterol and TGs levels and increased high-density lipoprotein (HDL) (101). Additionally, a prominent decrease in mean fasting insulin levels was observed. This finding is consistent with the potential improvement in insulin sensitivity (101). Daily administration of LY2405319 for 28 days resulted in a less atherogenic lipoprotein profile (13, 101, 109). However, in contrast to the glucose-lowering effect of LY2405319 in monkeys and mice, there were no statistically significant changes in human fasting glucose (4, 101).

PF-05231023, another FGF21 long-acting analog, considerably reduced body weight, plasma TGs and LDLs and increased HDLs and adiponectin in overweight or obese subjects with T2DM (13, 34). However, PF-05231023 did not show any significant effects on glycemic control (4, 34). Kim et al. showed pulse rate, systolic and diastolic blood pressure increased after administration of PF-05231023 in a dose- and time-related manner (110). Additionally, modest changes in bone absorption and resorption markers were observed during PF-05231023 administration (34, 110). Although it is not clear whether it is a direct effect of FGF21 or an indirect effect on bone turnover due to weight loss (111).

A PEGylated FGF21 analog called Pegbelfermin (formerly BMS-986036) was tested in biopsy-confirmed NASH patients previously (12). This study showed daily or weekly administration of Pegbelfermin reduced TG and LDL while increasing HDL and adiponectin (12, 13). Additionally, Pegbelfermin administration caused a significant reduction in hepatic fat content and a prominent decrease in plasma markers of liver injury [alanine aminotransferase (ALT) and aspartate aminotransferase (AST)] and fibrosis (4, 12). Another 12-week phase 2 study with the administration of Pegbelfermin in patients with obesity and T2DM demonstrated a remarkable increase in HDL and adiponectin levels but no statistically significant decrease in weight, fasting insulin and HbA1C levels were observed (103, 109). It is reported that Pegbelfermin induces anti-pegbelfermin and anti-FGF21 antibodies, which may cross-react with the endogenous FGF21 (80).

Efruxifermin, a long-acting Fc-FGF21 fusion protein was assessed in a trial for the treatment of NASH (104). This study showed that treatment with Efruxifermin for 12 weeks was associated with an absolute reduction in hepatic fat fraction and this reduction in hepatic fat content was followed by a rapid and marked decrease in liver stress and injury markers (such as ALT and AST and pro-C3) (13, 104). Reduced TG, LDL, fasting glucose and fasting insulin were reported in this study while increasing HDL and adiponectin levels were observed (104). Efruxifermin also improved glycemic control by a substantial reduction in HbA1C (13, 104).

BFKB8488A is a humanized antibody that specifically activates the FGFR1/β-Klotho complex (112). In phase 2 clinical trial consisting of NAFLD patients, 12 weeks of administration of BFKB8488A diminished liver fat, serum TG and pro-C3 while increasing adiponectin and HDL (13, 105, 112).

MK-3655, a monoclonal antibody targeted to β-klotho and FGFR1c, in a clinical trial in phase 2 showed improvements in glycemic control and reduction in liver fat content while decreasing serum TG and LDL (13, 105).

LLF580, a genetically engineered variant of human FGF21, demonstrated beneficial effects on serum lipids profile (caused a decrease in total cholesterol and LDL, and an increase in HDL level) in obese and mildly hypertriglyceridemic adults (107). This study also showed a substantial reduction in hepatic fat content and improvement in liver function tests and biomarkers of liver injury suggesting that LLF580 may be beneficial for the treatment of metabolic disorders such as NAFLD (107).

These clinical trials on FGF21 analogs and mimetics showed that they could be used as potential therapeutic agents for metabolic and liver disorders. Besides, safety concerns for instance cardiovascular side effects and possibility of bone loss raise the questions like will FGF21 analogs be safe in chronic treatments and how would potential side effects including anti-drug antibodies (82) influence the development of FGF21 analogs. Another challenging issue is FGF21 resistance which was discussed by Fisher et al. (113). According to their investigations, diet-induced obese mice had an elevated endogenous level of FGF21 and responded poorly to acute exogenous FGF21 administration (113). Also, a study on NAFLD model of mice revealed that expression level of β-klotho was negatively correlated with plasma FGF21, intrahepatic triglyceride and body weight which suggested a resistance to FGF21 (14). However, other authors did not find any evidences of FGF21 resistance in obese mice (114) and as mentioned above FGF21 analogs are able to decrease body weight, plasma lipids and improve insulin sensitivity in obese patients with no obvious evidence of FGF21 resistance (115). Admitting these facts, FGF21 resistance due to its co-receptor alteration is a controversial issue to be considered during the further investigations of FGF21 as a novel pharmacological agent (14).

Therefore, larger and long-term trials should be established in order to assess their safety and efficacy.

## Conclusion

Taken together our review suggested that FGF21 has a major role in the pathophysiology and treatment of AFLD, NAFLD and HCC. Administrating of FGF21 analogs and mimetics has demonstrated therapeutic benefit in human and rodent models of metabolic diseases, but still more studies and clinical trials will be required to prove the efficacy of these treatments. FGF21 is also used as a diagnostic biomarker for metabolic associated diseases of the liver. Also, despite the few studies that have been done on the genetic variations of FGF21, significant results have been obtained regarding the direct and indirect effects of these variations on metabolic diseases. In summary, the present study suggests that FGF21 administration can ameliorate fatty liver diseases and HCC furthermore studies are needed.

## Author contributions

All authors listed have made a substantial, direct, and intellectual contribution to the work and approved it for publication.

## Conflict of interest

The authors declare that the research was conducted in the absence of any commercial or financial relationships that could be construed as a potential conflict of interest.

## Publisher's note

All claims expressed in this article are solely those of the authors and do not necessarily represent those of their affiliated organizations, or those of the publisher, the editors and the reviewers. Any product that may be evaluated in this article, or claim that may be made by its manufacturer, is not guaranteed or endorsed by the publisher.
